# Nose-Only Water-Pipe Smoke Exposure in Mice Elicits Renal Histopathological Alterations, Inflammation, Oxidative Stress, DNA Damage, and Apoptosis

**DOI:** 10.3389/fphys.2020.00046

**Published:** 2020-02-11

**Authors:** Abderrahim Nemmar, Sumaya Beegam, Priya Yuvaraju, Javed Yasin, Badreldin H. Ali, Ernest Adeghate

**Affiliations:** ^1^Department of Physiology, College of Medicine and Health Sciences, United Arab Emirates University, Al Ain, United Arab Emirates; ^2^Zayed Center for Health Sciences, United Arab Emirates University, Al Ain, United Arab Emirates; ^3^Department of Internal Medicine, College of Medicine and Health Sciences, United Arab Emirates University, Al Ain, United Arab Emirates; ^4^Department of Pharmacology and Clinical Pharmacy, College of Medicine and Health Sciences, Sultan Qaboos University, Muscat, Oman; ^5^Department of Anatomy, College of Medicine and Health Sciences, United Arab Emirates University, Al Ain, United Arab Emirates

**Keywords:** water-pipe smoke, kidney injury, inflammation, oxidative stress, DNA damage, apoptosis

## Abstract

The prevalence of water-pipe tobacco smoking is increasing worldwide, and is relatively high among youth and young adults. Exposure to water-pipe smoke (WPS) has been reported to affect various systems including the respiratory, cardiovascular and reproductive systems. However, the impact of WPS exposure on the kidney has received only scant attention. Here, we assessed the effect of nose-only WPS exposure for one or four consecutive weeks on renal histology, inflammation, oxidative stress, DNA damage, and apoptosis. The duration of the session was 30 min/day and 5 days/week. Control mice were exposed to air. Light and electron microcopy analysis revealed that the WPS exposure (especially at 4-week time point) caused degeneration of the endothelial cells of the glomerular capillaries and vacuolar degenerations of the proximal convoluted tubules. WPS exposure also significantly decreased the creatinine clearance, and significantly increased proteinuria and urinary kidney injury molecule-1 (KIM-1) concentration. Kidney lipid peroxidation, reactive oxygen species, and oxidized glutathione were significantly increased. WPS exposure also affected the concentration of reduced glutathione and the activity of catalase. Likewise, renal concentrations of interleukin (IL)-6, IL-1β and KIM-1 were augmented by WPS exposure. Moreover, WPS caused DNA damage as evaluated by comet assay, and increased the expression of cleaved caspase-3 and cytochrome C in the kidney. We conclude that exposure of mice to WPS caused renal histopathological alterations, inflammation, oxidative stress, DNA damage, and apoptosis. If the latter findings could be substantiated by controlled human studies, it would be an additional cause for disquiet about an established public health concern.

## Introduction

The consumption of tobacco is considered to be a major public health problem causing substantial morbidity and mortality. In 2015, 6.4 million deaths were attributable to tobacco smoking worldwide, corresponding to a 4.7% rise in smoking-attributable deaths since 2005 (GBD 2015 Tobacco Collaborators, 2017). Cigarette smoking (CS) is the most prevalent method of tobacco usage (GBD 2015 Tobacco Collaborators, 2017). It is well known that CS affects multiple organ systems resulting in pulmonary and extrapulmonary adverse effects (GBD 2015 Tobacco Collaborators, 2017). In this context, it is well-established that CS is responsible for most of lung cancer cases and is the major risk factor for the development of chronic obstructive pulmonary disease, pulmonary edema, recurrent infections, and cardiovascular diseases (GBD 2015 Tobacco Collaborators, 2017). Moreover, data from a meta-analysis of prospective cohort studies showed that CS was linked with an increased risk of kidney disease in the general population (Xia et al., 2017). The latter findings were independent of well-known risk factors for kidney disease such as age, hypertension, diabetes mellitus, and body mass index (Xia et al., 2017).

Water-pipe smoking (also called *hookah*, *shisha*, Hubble bubble, and *narghila*) is a method of smoking that uses tobacco sweetened with either fruit or molasses sugar, which makes the smoke more aromatic than CS. The tobacco in water-pipe smoking is exposed to high heat from burning charcoal, and the generated smoke has an equivalent or higher toxicity than cigarette smoke (Centers for Disease Control and Prevention, 2016). Several reports showed that water-pipe smoking prevalence is increasing worldwide, and is particularly high among young adult population (Akl et al., 2013; Sidani et al., 2018).

We have recently demonstrated that exposure to water-pipe smoke (WPS) in mice causes impairment of lung function, thrombotic events, and triggers inflammation and oxidative stress in various organs including the lung, heart, and testes (Nemmar et al., 2013c,d, 2016a, 2017a; Ali et al., 2017). While some studies have reported negative health effects of CS on the kidney (Xia et al., 2017), little is known about the potential adverse impact of WPS exposure on the kidney. It has been recently reported that exposure to WPS causes oxidative stress in the kidney (Rababa’h et al., 2016). However, the mechanisms underlying this effect remain unknown. Therefore, the aim of the present work is to investigate the mechanism of actions of nose-only WPS on the kidneys, and its time course (1 week and 4 weeks). This included a study of renal function and histology assessed by light and electron microscopy, inflammation, oxidative stress, DNA damage, and apoptosis.

## Materials and Methods

### Animals and WPS Exposure

This project was reviewed and approved by the Institutional Review Board of the United Arab Emirates University, College of Medicine and Health Sciences, and experiments were performed in accordance with protocols approved by the Institutional Animal Care and Research Advisory Committee.

BALB/c mice (Taconic Farms Inc., Germantown, NY, United States) were housed in a conventional animal house and maintained on a 12-h light-dark cycle (lights on at 6:00 am). The animals were placed in cages and supplied with pelleted food and water *ad libitum*. Following 1 week of acclimatization, animals were randomly divided into air (control) and WPS-exposed groups.

Mice were placed in soft restraints and connected to the exposure tower (Nemmar et al., 2013c, d, 2015c, 2016a, 2017a). The animals were exposed to either WPS or air through their noses using a nose-only exposure system connected to a water-pipe (InExpose System, Scireq, Canada). Animals were exposed to a commercially available honey-flavored tobacco (Al Fakher Tobacco Trading, Ajman, United Arab Emirates). Tobacco was lit with an instant light charcoal disk (Star, 3.5 cm diameter and 1 cm width). As is the case for human use, the smoke from the water-pipe passes first through the water before it is drawn into the exposure tower. The exposure regimen is controlled by a computerized system (InExpose System, Scireq, Canada). A computer-controlled puff was generated every minute, leading to a 2 s puff duration of WPS exposure followed by 58 s of fresh air. The duration of an exposure session was 30 min/day (Nemmar et al., 2013c, d, 2015c, 2016a). This was selected from a published work that has assessed the cardiorespiratory effects of WPS in healthy subjects (Hakim et al., 2011). Mice were exposed for 1 week or 4 weeks either to air or WPS.

Immediately after the last exposure session to WPS or air, mice were placed in metabolic cages, and urine of each mouse was collected over a 24-h period and the volume measured. Immediately after urine collection, mice were anesthetized with sodium pentobarbital (45 mg/kg, i.p.), and blood was drawn from the inferior vena cava in ethylenediaminetetraacetic acid (4%). The collected blood was centrifuged at 4°C for 15 min at 900 × *g*, and the plasma samples were stored at −80°C pending analysis.

The animals were then sacrificed with an overdose of anesthesia. The kidneys from all mice were collected and rinsed with ice-cold PBS (pH 7.4). The kidneys were weighed and the right kidney was immediately frozen at −80°C until biochemical and molecular studies whereas left kidney was used for morphological studies.

### Light Microscopy

Left kidney from mice, which were exposed to air or WPS for 1 or 4 weeks, were collected and fixed with 10% formaldehyde. Seven-micrometer sections were cut from the paraffin blocks and stained with Periodic acid-Schiff. The sections were later examined according to a previously published method (Ali et al., 2010; Nemmar et al., 2010).

### Electron Microscopy

Small fragments (2∼ mm^3^) of kidney samples of mice were trimmed of connective tissue and fixed in Karnovsky’s fixative, and processed for electron microscopy according to a previously described method (Karnovsky, 1965; Adeghate, 1998). Briefly, kidney tissue samples of mice were washed three times in sodium cacodylate buffer and post-fixed for 1 h in 1% osmium tetroxide solution at room temperature. The tissue samples were rinsed five times in buffer after post-fixation. The samples were dehydrated in ethanol, cleared in propylene oxide and subsequently embedded in epon. 0.5 μm thick sections were made with Reichert Ultracut S (Reichert Inc., NY, United States) after polymerization and stained with toluidine blue. Selected area of the resin block was trimmed for ultrathin sections. 80 nm thin sections were made with diamond knife, placed on copper grids and counterstained with lead citrate and uranyl acetate. The grids were examined with FEI Tecnai^TM^ transmission electron microscope (FEI, Hillsboro, OR, United States).

### Biochemical Analysis

Right kidneys from the two groups of mice were homogenized as described before (Nemmar et al., 2015b, 2017b). The homogenates were centrifuged for 10 min at 3000 × *g* to remove cellular debris, and the supernatants were used for further analysis (Nemmar et al., 2015b). Protein content was measured by Bradford’s method. NADPH-dependent membrane lipid peroxidation (LPO) was measured as thiobarbituric acid reactive substance using malonedialdehyde as standard (Sigma-Aldrich Fine Chemicals, St. Louis, MO, United States) (Raza et al., 2004). Reactive oxygen species (ROS) were measured in kidney homogenates, using 2′,7′-dichlorofluorescein diacetate (Molecular Probes, Eugene, OR, United States) as a fluorescent probe as previously described (LeBel et al., 1990; Nemmar et al., 2013c, 2015a). The results were normalized as ROS produced per milligram of protein. Measurement of GSH concentrations was carried out in control and WPS-exposed animals according to method described by commercially available kit (Sigma-Aldrich Fine Chemicals, Germany). Measurements of oxidized glutathione [glutathione disulfide (GSSG)] and catalase activity were carried out in control and WPS-exposed animals using spectrophotometric method with commercially available kits (Cayman Chemical, Ann Arbor, MI, United States).

The concentrations of kidney injury molecule-1 (KIM) (Duo Set, R & D systems, Minneapolis, MN, United States) in kidney homogenates and in urine, and the kidney homogenate concentrations of interleukin-6 (IL-6) (Duo Set, R & D systems, Minneapolis, MN, United States), and IL-1β (Duo Set, R & D systems, Minneapolis, MN, United States) were determined using commercial Kits. The concentrations of cytochrome C in kidney homogenate were quantified using commercial Kit purchased from R & D systems (Duo Set, Minneapolis, MN, United States).

The concentrations of creatinine in urine and plasma and that of protein in urine were spectrophotometrically measured using commercial kits (BioMerieux, Marcy-l’Etoile, France).

### Assessment of DNA Damage by COMET Assay

In separate animals, immediately after sacrifice, kidneys were removed from each animal. Single-cell suspensions of the different kidneys were obtained and analyzed according to the method described in our previous publications (Al Suleimani et al., 2016; Nemmar et al., 2016a,b,c). Each collected kidney was washed in a chilled medium (RPMI 1640, 15% DMSO, 1.8% (w/v) NaCl). The kidney tissues were put in 1.5 ml medium and cut finely into pieces in a Petri dish. The slices were allowed to deposit and the supernatant was collected in a 15 ml tube. The collected cell suspension was centrifuged at 1000 rpm for 5 min at 4°C. The supernatant was removed and the pellets were suspended in 0.5 ml of the medium. The cell suspensions were mixed with low melting point agarose solution (0.65%) and spread onto agarose (1.5%)–precoated microscope slides. For each group, five slides were prepared and incubated in ice cold lysis buffer (2.5M NaCl, 10mM Tris, 100mM EDTA, 1% Triton X-100 and 10% DMSO) at 4°C for at least 1 h to remove the cell membranes. Following incubation, slides were placed in a horizontal electrophoresis unit and incubated in electrophoresis buffer (0.2M EDTA, 5M NaCl, pH 10) for 20 min for DNA unwinding and the expression of alkali labile sites. Then, electrophoresis was conducted for 20 min at 25V and 300 mA. After that, the slides were neutralized with Tris buffer (0.4M Trizma base, pH 7.5) for 5 min and washed with methanol. Then the slides were stained with propidium iodide, as previously described (Olive et al., 1994; Nemmar et al., 2016c). All these steps were performed in darkness to prevent additional DNA damage. The slides were mounted on a fluorescent microscope and cell scoring was performed. The measurement of length of the DNA migration (i.e., diameter of the nucleus plus migrated DNA) was calculated using the image analysis Axiovision 3.1 software (Carl Zeiss, Canada) (Hartmann and Speit, 1997; Nemmar et al., 2016c).

### Western Blot Analysis for the Detection of Cleaved Caspase-3

Protein expressions for cleaved caspase-3 were measured using Western blotting technique (Nemmar et al., 2018). Kidney tissues harvested from the mice were snap frozen immediately with liquid nitrogen and stored at −80°C. Later, the tissues were weighed, rinsed with saline and homogenized with lysis buffer (pH 7.4) containing NaCl (140 mM), KCl (300 mM), trizma base (10 mM), EDTA (1 mM), Triton X-100 0.5% (v/v), sodium deoxycholate 0.5% (w/v), protease, and phosphatase inhibitor. The homogenates were centrifuged for 20 min at 4°C. The supernatants were collected and protein estimation was made using a Pierce bicinchoninic acid protein assay kit (Thermo Scientific). A 35 μg sample of protein was electrophoretically separated by 10% sodium dodecyl sulfate polyacrylamide gel electrophoresis and then transferred onto polyvinylidene difluoride membranes. The immunoblots were then blocked with 5% non-fat milk and subsequently probed with rabbit monoclonal cleaved caspase-3 antibody (1:250 dilution, Cell Signalling Technology) at 4°C overnight. The blots were then incubated with goat anti-rabbit IgG horseradish peroxidase conjugated secondary antibody (1:5000 dilution, Abcam) for 2 h at room temperature and developed using Pierce enhanced chemiluminescent plus Western blotting substrate Kit (Thermo Scientific). The densitometric analysis of the protein bands was performed with Typhoon FLA 9500 (GE Healthcare Bio-Sciences AB, Uppsala, Sweden). Blots were then re-probed with mouse monoclonal GAPDH antibody (1:5000 dilution, Abcam) and used as a control.

### Statistics

All statistical analyses were performed with GraphPad Prism Software version 7. To determine whether parameters were normally distributed, the Shapiro–Wilk normality test was applied. Normally distributed data were analyzed using the unpaired *t*-test for differences between the two groups. Non normally distributed data (cleaved caspase-3) were analyzed using the Mann–Whitney test for differences between groups. All the data in figures and table were reported as mean ± SEM. *P* values < 0.05 are considered significant.

## Results

### Effect of WPS on Kidney Histology Assessed by Light and Electron Microscopy

**FIGURE 1:**
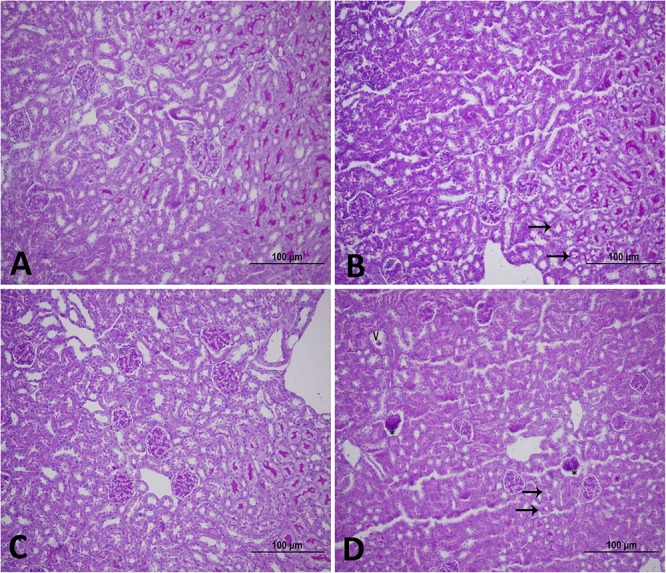
Representative light [Periodic acid-Schiff (PAS) staining] micrographs of kidney tissues of mice at the end of 1-week exposure period to air (control, **A**), 1-week exposure period water pipe smoke (WPS) exposure **(B)**, 4-week exposure period to air (control, **C**) or 4-week exposure period to WPS **(D)**. Note that the kidney sections of mice exposed to WPS showed vacuoles (V), glomerular degeneration (^∗^), and loss of loss of PAS staining (arrows). Scale bar = 100 μm; *n* = 12.

Figure 1 shows the effect of WPS or air exposure on kidney morphology assessed by light microscopy at the end of 1-week or 4-week time point. The histopathological changes caused by WPS exposure were most evident at 4-week time point. The latter manifested as vacuoles and glomerular degeneration at the light microscopy level (Figures 1B,D).

**FIGURE 2:**
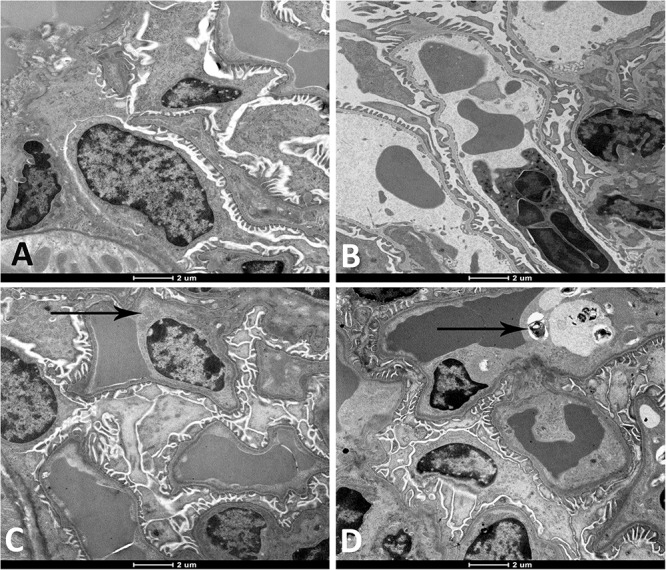
Representative electron micrographs of the glomerular structures of the kidney of mice at the end of 1-week exposure period to air (control, **A**), 1-week exposure period water pipe smoke (WPS) exposure **(B)**, 4-week exposure period to air (control, **C**) or 4-week exposure period to WPS **(D)**. Degenerating endothelial cells (black arrow shown in micrograph **D**) was discerned in the kidney of mice exposed to WPS at 4-week exposure period. Scale bar = 2 μm; *n* = 10.

As illustrated in Figure 2, the electron microscopic analysis of the kidney following exposure to WPS revealed the presence of degeneration of the endothelial cells of the glomerular capillaries which was seen only at 4-week time point (Figure 2D).

**FIGURE 3:**
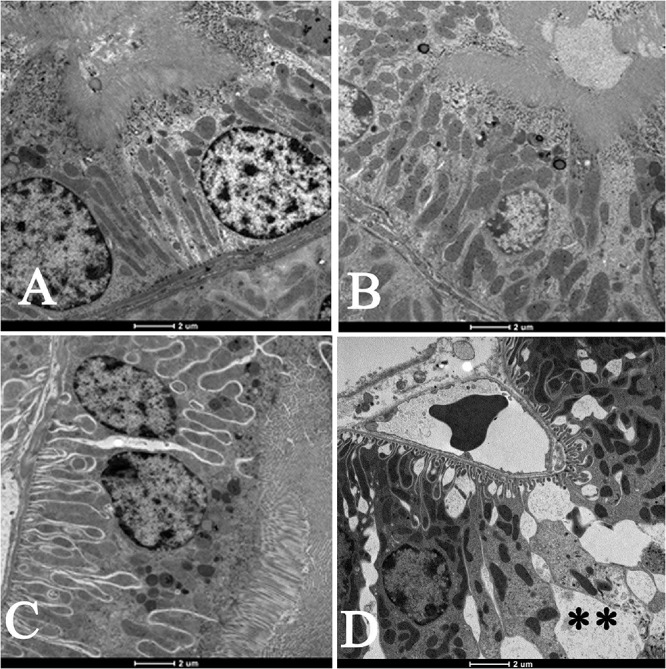
Electron micrographs of the proximal convoluted tubules (PCT) of the kidney of mice at the end of 1-week exposure period to air (control, **A**), 1-week exposure period water pipe smoke (WPS) exposure **(B)**, 4-week exposure period to air (control, **C**) or 4-week exposure period to WPS **(D)**. Large vacuoles (^∗∗^) were observed in the wall of the PCT of the kidney of mice exposed to WPS at 4-week exposure period. Scale bar = 2 μm; *n* = 10.

Moreover, as shown in Figures 3A–D, compared with air-exposed group, WPS exposure for 4 weeks induced a severe vacuolar degeneration of the proximal convoluted tubules (Figure 3D).

### Effect of WPS on the Creatinine Clearance, Proteinuria, and KIM-1 Concentrations in the Urine

**FIGURE 4:**
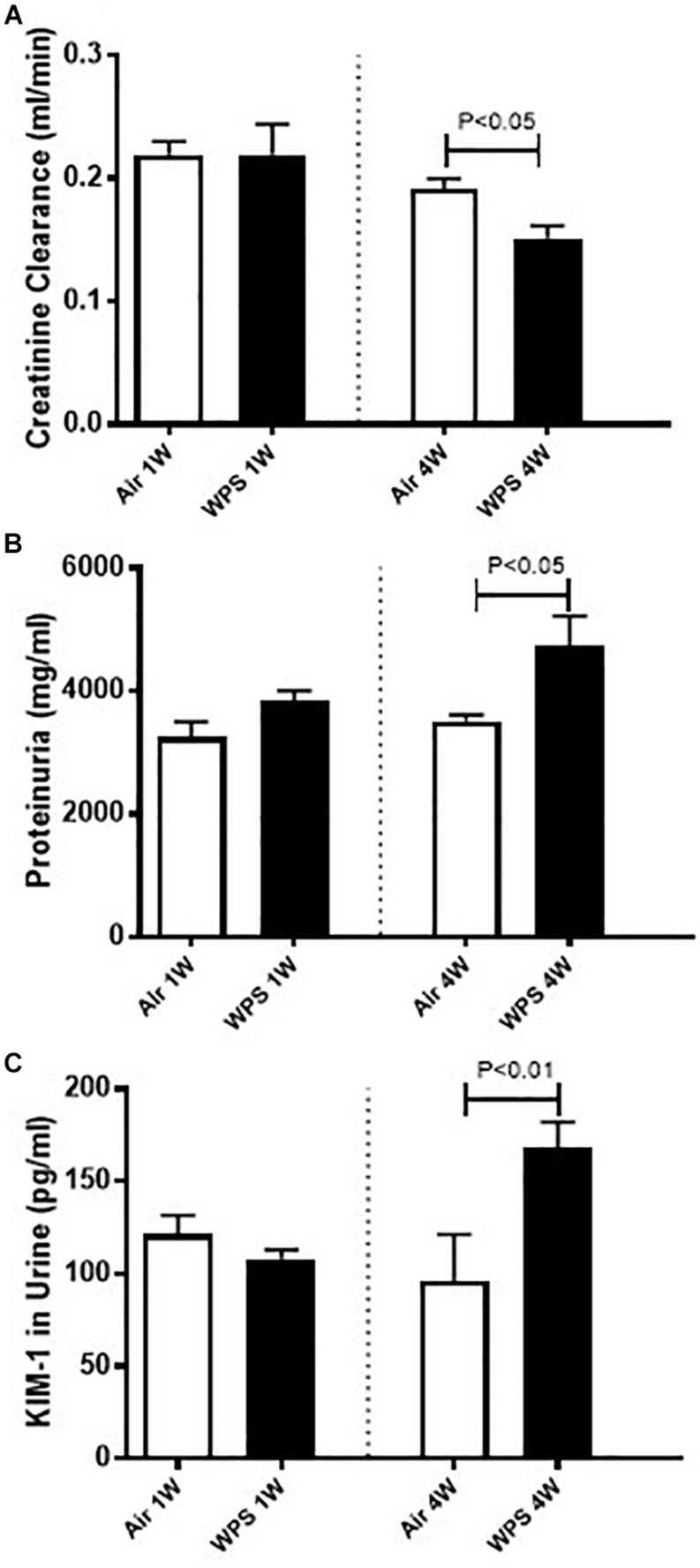
Creatinine clearance **(A)**, and urinary concentrations of proteins **(B)** and kidney injury molecule-1 (KIM-1) **(C)** in mice at the end of the 1-week and 4-week exposure periods to air (control) or water-pipe smoke (WPS). Data are mean ± SEM (*n* = 5–6).

Figure 4 shows that at 1-week time point, neither the creatinine clearance, nor the proteinuria, or the KIM concentration in the urine were affected by WPS exposure. However, at the end of 4 weeks of exposure, the creatinine clearance was significantly reduced in WPS group (*P* < 0.05) compared with air group. Additionally, the proteinuria (*P* < 0.05) and the urinary concentration of KIM-1 (*P* < 0.01) were both significantly increased in WPS group at 4-week time point compared with their respective control groups.

### Effect of WPS on the Renal Levels of LPO, ROS, GSSG, GSH, and Catalase

**FIGURE 5:**
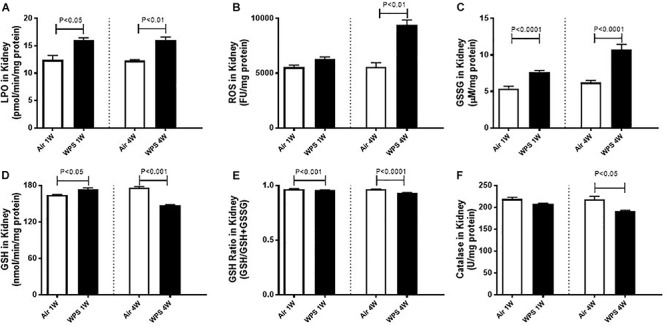
Kidney homogenate levels of lipid peroxidation (LPO, **A**), reactive oxygen species (ROS, **B**), oxidized glutathione (GSSG, **C**), reduced glutathione (GSH, **D**), glutathione ratio (GSH/GSH+GSSG, **E**), and catalase (**F**) in mice at the end of the 1-week and 4-week exposure periods to air (control) or water-pipe smoke (WPS). Data are mean ± SEM from three independent experiments.

Figure 5 depicts the effect of WPS exposure on some markers of oxidative stress in kidney homogenates. Compared with their respective control groups, WPS exposure for both 1 week (*P* < 0.05) and 4 weeks (*P* < 0.01) induced a significant increase in the concentration of LPO (Figure 5A).

There was no significant increase in the levels of ROS at 1-week time point in WPS-exposed group compared with air group (Figure 5B). However, at 4-week time point, the levels of ROS were markedly increased by WPS exposure compared with air group (*P* < 0.01; Figure 5B).

Likewise compared with their respective control groups, WPS exposure for both 1 week and 4 weeks induced a significant increase (*P* < 0.0001) in the levels of oxidized glutathione, GSSG (Figure 5C).

At 1-week exposure period, the concentration of the reduced GSH was slightly but significantly increased by WPS compared with air-exposed group (*P* < 0.05; Figure 5D). However, at 4-week exposure period, the concentration of GSH was significantly decreased by WPS exposure (*P* < 0.001; Figure 5D).

The assessment of the glutathione redox status as a function of GSH/GSH + GSSG which provides a direct measure of oxidative stress indicates a statistically significant decrease of this ratio in WPS groups at both 1 week (*P* < 0.001) and 4 weeks (*P* < 0.0001). The decrease of this ratio indicates an increase in oxidative stress.

Figure 5F shows that at 1-week time point, and compared with air-exposed mice, catalase activity was not significantly affected by WPS exposure. However, at 4-week time point, the activity of catalase was significantly decreased by WPS exposure (*P* < 0.05; Figure 5E).

### Effect of WPS on the Concentrations of KIM-1, IL-6, and IL-1β in the Kidney

**FIGURE 6:**
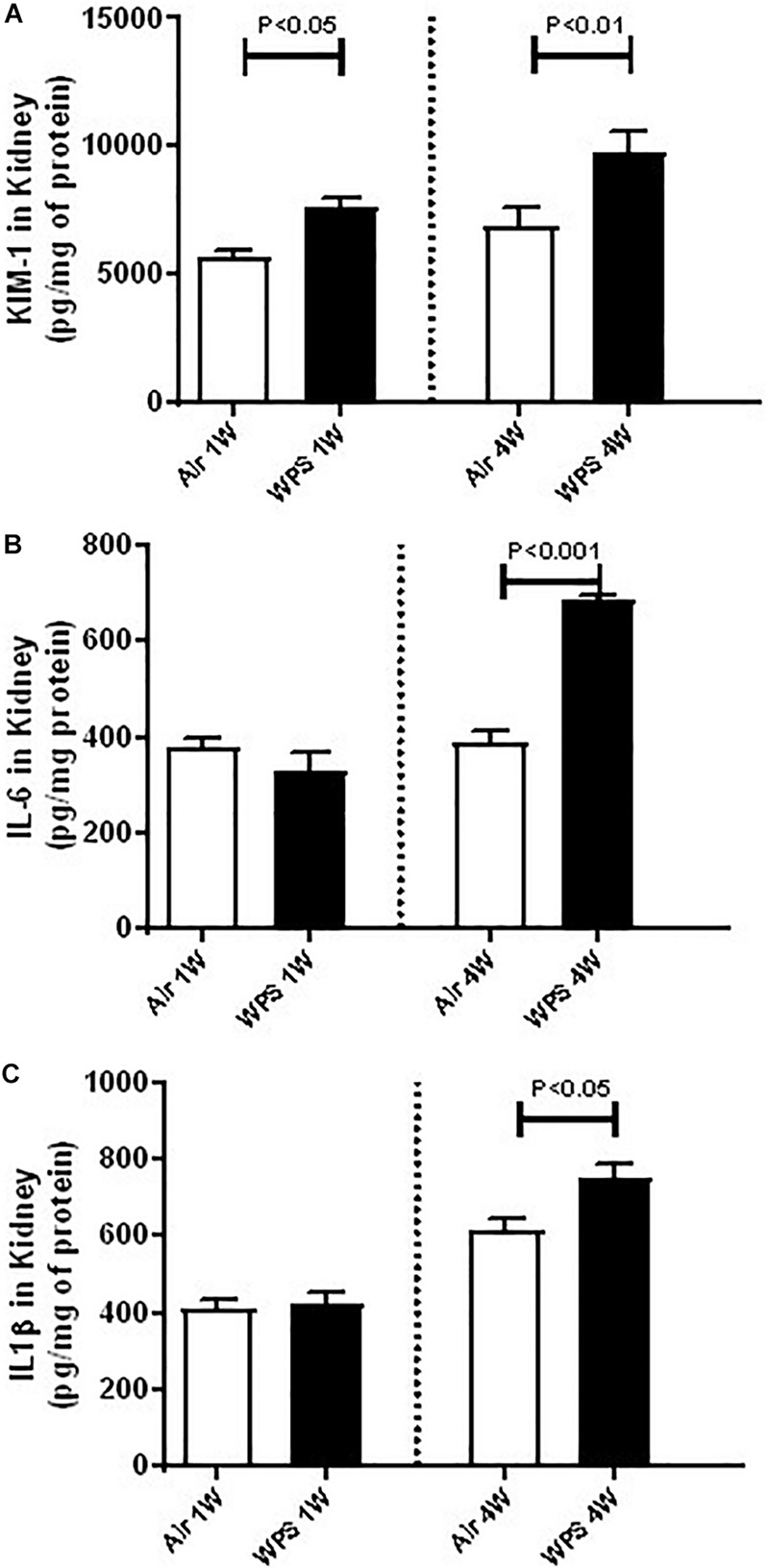
Kidney homogenate concentrations of kidney injury molecule-1 (KIM-1, **A**), interleukin-6 (IL-6, **B**), and IL-1β **(C)** in mice at the end of the 1-week and 4-week exposure periods to air (control) or water-pipe smoke (WPS). Data are mean ± SEM (*n* = 5–8).

Figure 6A shows that mice exposed to WPS showed a significantly higher concentration of KIM-1 in the kidney homogenates at both 1-week (*P* < 0.05) and 4-week (*P* < 0.01) time points.

Compared with control group, the concentrations of IL-6 and IL-1β were unaffected by WPS exposure at 1-week time point. Nevertheless, at 4-week time point, the concentrations of IL-6 (*P* < 0.001; Figure 6B) and that of IL-1β (*P* < 0.05; Figure 6C) were both significantly elevated by WPS exposure, compared to their respective controls.

### Effect of WPS on Kidney DNA Damage

**FIGURE 7:**
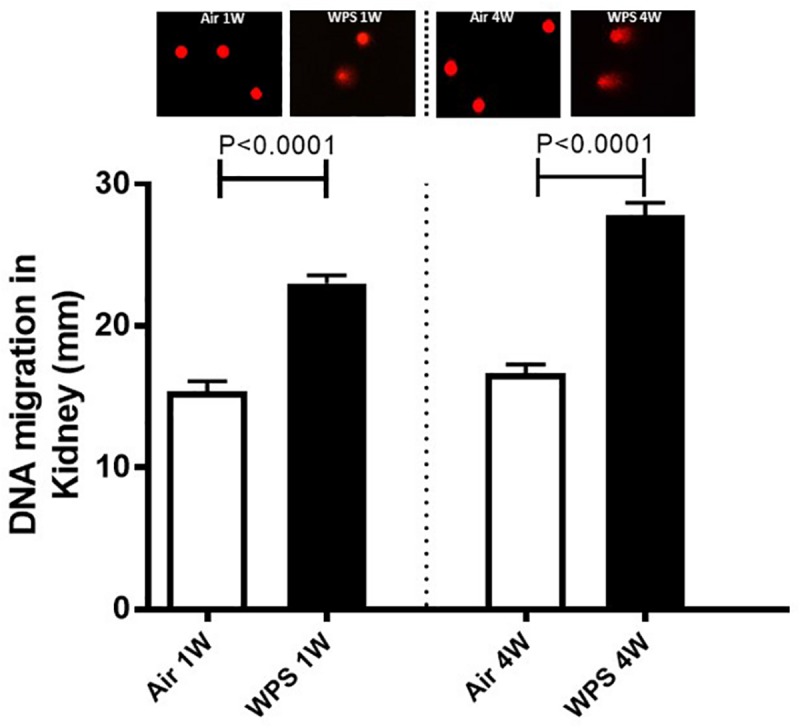
DNA migration (mm) in the kidney tissues assessed by Comet assay in mice at the end of the 1-week and 4-week exposure periods to air (control) or water-pipe smoke (WPS). Images illustrating the quantification of the DNA migration by the Comet assay under alkaline conditions in mice at the end of the 1-week and 4-week exposure periods to air or WPS. Data are mean ± SEM (*n* = 5).

Figure 7 displays the time-effect of WPS on DNA damage in the kidney assessed by Comet assay. Compared with their respective control groups, exposure to WPS for either 1 or 4 weeks induced a significant increase in DNA damage (*P* < 0.0001).

### Effect of WPS on the Expression of Cleaved Caspase-3 in the Kidney

**FIGURE 8:**
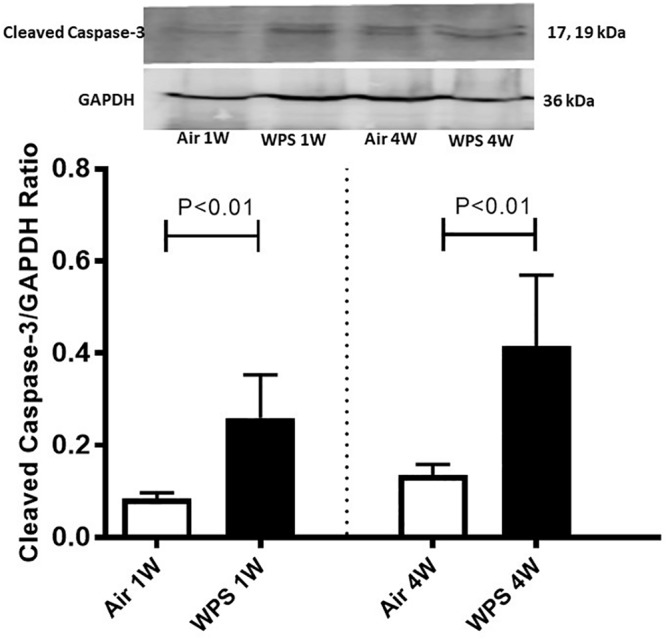
Western blot analysis and graphic representation of cleaved caspase-3 protein levels in the kidney tissues of mice at the end of the 1-week and 4-week exposure periods to air (control) or water-pipe smoke (WPS). Data are mean ± SEM (*n* = 6).

Western blot for the detection of cleaved caspase-3 is represented in Figure 8 and Supplementary Figure S1. Compared with the control group, the exposure of mice to WPS for either 1 or 4 weeks caused a significant increase in the expression of cleaved caspase-3 (*P* < 0.01).

### Effect of WPS on the Concentration of Cytochrome C in the Kidney

**FIGURE 9:**
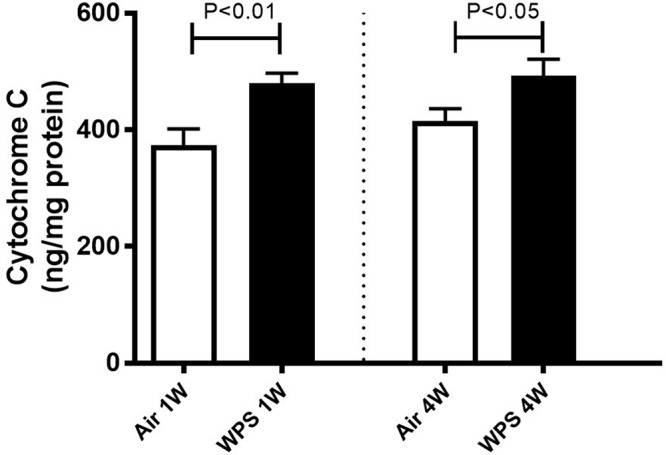
Kidney homogenate concentrations of cytochrome C in mice at the end of the 1-week and 4-week exposure periods to air (control) or water-pipe smoke (WPS). Data are mean ± SEM (*n* = 7–8).

As shown in Figure 9, compared with the control group, the exposure of mice to WPS for either one (*P* < 0.01) or four (*P* < 0.05) weeks induced a significant elevation in the concentration of cytochrome C in the kidney homogenates.

## Discussion

In this work, we showed that exposure to WPS in mice caused biochemical, histopathological and molecular kidney changes, including degeneration of the endothelial cells of the glomerular capillaries and vacuolar degeneration of the proximal convoluted tubules, oxidative stress, inflammation, DNA damage, and apoptosis.

It is well known that the kidneys receive 1/5th of the cardiac output, where a significant amount of blood is filtered and toxic substances from pollution are concentrated (Xu et al., 2018). The latter makes the kidneys particularly susceptible to environmental pollution (Xu et al., 2018). In fact, diseases of the kidneys are being acknowledged as a major public health problem as their prevalence is continuously increasing reaching as high as 10% of certain populations (Stenvinkel, 2010).

Cigarette smoking is considered as an independent risk factor for the development of kidney disease not only in patients with diabetes mellitus but also in the general population (Xia et al., 2017). The latter effect could be explained by the cross-talk between the lung (the route of entry of CS) and the kidney (Domenech et al., 2017). In fact, under pathophysiological situations, it has been reported that kidney function can be altered by chemical mediators released from the lung which can translocate across the alveolar-capillary barrier and reach the systemic circulation (Nemmar et al., 2013a; Domenech et al., 2017; Xu et al., 2018).

It has been shown that water-pipe tobacco smoking is associated with considerable toxicant exposure. In fact, compared with to a single cigarette, a single session of water-pipe use is accompanied with 1.7 times increase in the nicotine, 8.4 times rise in the CO, and 36 times increase in the tar (Cobb et al., 2010). Experimental studies have shown that pulmonary exposure to cerium oxide nanoparticles or CS induce oxidative stress in the kidneys (Nemmar et al., 2013b, 2017b). The data on the effects of WPS on the kidneys is very scarce. It has been recently reported that whole-body WPS exposure in mice induces oxidative stress and elevates urea and creatinine (Rababa’h et al., 2016). However, no study has comprehensively assessed the nose-only effects of WPS on the kidney and the mechanisms underlying these effects. The latter mode of exposure to WPS better represents human exposure scenario as it avoids the ingestion of nicotine or tar substances by animals when cleaning their fur (Rinaldi et al., 2012).

Our data obtained by light and electron microscopy analysis showed that WPS exposure for four consecutive weeks induced kidney morphological changes characterized by degeneration of the endothelial cells of the glomerular capillaries and vacuolar degenerations of the proximal convoluted tubules. Such effects have never been reported before. Along with the histological alterations, we also found that nose-only WPS exposure for 4 weeks caused a significant reduction of creatinine clearance which is commonly used to estimate glomerular filtration rate. The latter finding is suggestive of a decline in renal function (McMahon and Waikar, 2013; Veuthey et al., 2014). Moreover, at 4-week exposure period to WPS, we found a significant increase of proteinuria. Proteinuria has long been acknowledged as a result of kidney injury, and measurement of urinary protein is an important component for monitoring kidney diseases (McMahon and Waikar, 2013). Our data show that the urinary concentration of KIM-1 was significantly augmented by WPS exposure at 4-week time point. KIM-1 is a relatively novel and very sensitive biomarker of renal injury, and urinary KIM-1 concentrations have been reported to correlate with proteinuria (Waring and Moonie, 2011; McMahon and Waikar, 2013). Using whole-body exposure system, it has been shown that serum creatinine was increased at the end of 6 days exposure to WPS (Rababa’h et al., 2016). However, at 30 days time point, both blood urea nitrogen and serum creatinine were elevated by WPS exposure (Rababa’h et al., 2016).

Oxidative stress happens when excess oxygen radicals are generated in cells, which might overcome the normal cellular antioxidant capacity. When the concentrations of ROS are not controlled by internal defense mechanisms such as antioxidants (e.g., glutathione) or enzymes involved in oxygen radical scavenging (e.g., catalase), oxidative injury will occur and cause damage to cell membranes and other structures including proteins, lipids, lipoproteins, and DNA, which could lead to cytotoxicity and genotoxicity (Birben et al., 2012). In order to outline the mechanisms underlying the renal injury induced by WPS, we have quantified markers of oxidative stress including LPO, ROS, GSSG, and the reduced GSH and the antioxidant enzyme catalase in kidney homogenates. Our data show that WPS exposure induced a significant increase in the levels of LPO at one and 4-week time points and ROS at 4 weeks, indicating the occurrence of oxidative stress in the kidney. The latter effects were confirmed by a significant increase in the levels of oxidized glutathione, GSSG at one and 4-week time points. Moreover, at 1-week time point reduced GSH was slightly but significantly increased, conversely, catalase was not significantly affected. However, at 4-week time point both GSH and catalase were significantly reduced by WPS exposure. Moreover, the measurement of the glutathione ratio (GSH/GSH+GSSG) which provides a direct measure of oxidative stress indicates a statistically significant decrease of this ratio in WPS groups at both 1 week and 4 weeks. The latter indicates an increase in oxidative stress. An increase of antioxidants indicates an ongoing compensatory process to counterbalance the potentially damaging action of oxygen radicals induced by WPS (Nemmar et al., 2013b). However, a decrease of antioxidants observed at 4-week exposure period indicates that, as a consequence of oxidative stress, the studied antioxidants were consumed by a prolonged period of exposure to WPS (Nemmar et al., 2017b). Using whole-body exposure system, it has been reported that exposure to WPS induce an elevation of LPO in the kidney at 30-days but not at 6-days time point. In agreement with our findings, catalase was also found to decrease at only 30-days time point (Rababa’h et al., 2016). However, GSH concentrations were diminished but the decline did not reach statistical significance (Rababa’h et al., 2016).

Inflammation and oxidative stress are interconnected in several pathophysiological conditions, including those affecting the kidneys (Biswas, 2016). It is well-documented that ROS can induce cellular damage and initiate inflammation (Biswas, 2016). We have previously reported that exposure to WPS for 1 month induced oxidative stress and an increase of IL-6 and tumor necrosis factor α concentrations in heart homogenates (Nemmar et al., 2013d). However, the occurrence of inflammation in the kidney following WPS has not been studied before. Consequently, to further investigate the mechanism by which WPS induce kidney injury, we measured in kidney homogenates the concentrations of two pro-inflammatory cytokine, namely IL-6 and IL-1β, and KIM-1. KIM-1 which is known to be a sensitive and early marker of kidney damage was found to be significantly increased at 1-week time points. At 4-week exposure period to WPS, KIM-1, and both IL-6 and IL-1β were found to be significantly increased, indicating the occurrence of inflammation in the kidney. The increase in the level of KIM is supported by PAS-staining, which shows a reduced brush border in proximal convoluted tubules at 4-week time point.

Our data show for the first time the occurrence of DNA damage assessed by Comet assay in the kidney following the exposure to WPS. The latter effects could be ascribed to kidney inflammation and oxidative stress caused by WPS exposure. In fact, it is well-known that ROS may oxidize DNA or impede the mechanisms of DNA repair (Federico et al., 2007). It has been recently reported that chronic exposure to WPS causes DNA damage in the lung and heart (Nemmar et al., 2016a, 2017a). It is well-known that DNA injury can lead to apoptosis. Caspases are crucial mediators of apoptosis, and caspase-3 is a member of the caspase enzyme family, which holds a fundamental role in the implementation of apoptosis (Savitskaya and Onishchenko, 2015). Furthermore, we found a significant increase in the concentration of cytochrome C in kidney homogenates. It has been demonstrated that exposure of mice to WPS induces oxidative stress leading to an elevation in cytochrome C release in testicular and heart tissue homogenates suggesting mitochondrial damage (Nemmar et al., 2017c; Ali et al., 2019). In addition, it has been shown that the cytokine tumor-necrosis factor α triggers cytochrome C release of from the mitochondria to the cytoplasm, which is known to attach with the adaptor apoptotic protease activator factor-I and recruit caspase-9/3 that eventually result in cellular apoptosis (Yu et al., 2014; Savitskaya and Onishchenko, 2015). Here, we show that WPS exposure causes a significant increase in the expression of cytochrome C and cleaved caspase-3 in the kidney, confirming the occurrence of apoptosis. DNA damage and apoptosis have been reported to take place in adenine-induced kidney injury in mice (Ali et al., 2013).

We conclude that exposure to WPS induced renal histopathological alterations, inflammation, oxidative stress, DNA damage, and apoptosis. Our data provided mechanistic evidences for the injurious effect of WPS on the kidney. Additional studies on the mechanisms and the effect of WPS for longer durations of time, and identification of the constituents in WPS responsible for the observed nephrotoxicity (including the flavorings) are needed.

## Data Availability Statement

All datasets generated for this study are included in the article/Supplementary Material.

## Ethics Statement

The animal study was reviewed and approved by the Institutional Review Board of the United Arab Emirates University, College of Medicine and Health Sciences.

## Author Contributions

All authors have read and approved the manuscript. AN designed, planned, and supervised the experiments and wrote the manuscript. SB, PY, and JY performed the experiments. BA contributed in the design of the study and the writing of the manuscript. EA performed, analyzed, and interpreted the histology and electron microscopy parts of the work and contributed in writing of the manuscript.

## Conflict of Interest

The authors declare that the research was conducted in the absence of any commercial or financial relationships that could be construed as a potential conflict of interest.
