# Supplementary figures and images for: Nose-Only Water-Pipe Smoke Exposure in Mice Elicits Renal Histopathological Alterations, Inflammation, Oxidative Stress, DNA Damage, and Apoptosis
Source: Front Physiol. 2020 Feb 11;11:46. doi: 10.3389/fphys.2020.00046 (PMC7026484; doi:10.3389/fphys.2020.00046)

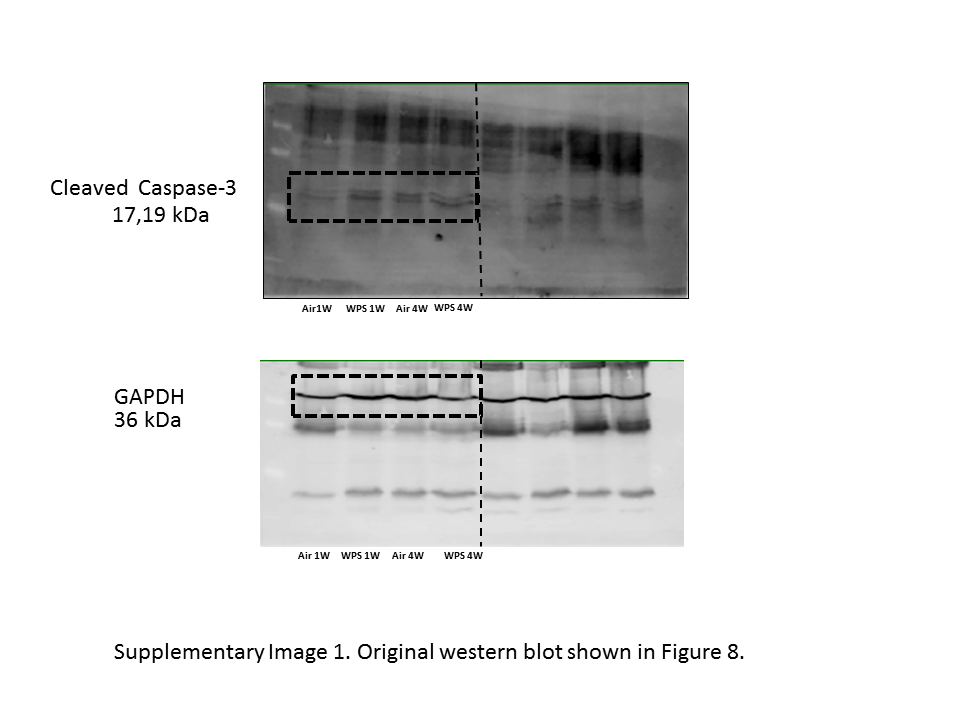

Supplement: Supplementary file 1 [file Image_1.TIF]
